# Case Report: Endoscopic full-thickness resection using dual and IT knives for a false gastric diverticulum and systematic review

**DOI:** 10.3389/fmed.2026.1938681

**Published:** 2026-09-09

**Authors:** Qiuchen Huang, Yongqi Li, Xun Xiao

**Affiliations:** 1 School of Medicine, University of Electronic Science and Technology of China, Chengdu, China; 2 Department of Gastroenterology and Hepatology, Sichuan Provincial People's Hospital, University of Electronic Science and Technology of China, Chengdu, China

**Keywords:** abdominal pain, case report, endoscopic full-thickness resection (EFTR), endoscopic resection, gastric diverticulum, medication-refractory

## Abstract

Gastric diverticula are the rarest gastrointestinal diverticula, traditionally managed medically or surgically. We report a case of a long-standing gastric diverticulum with postprandial abdominal pain refractory to acid suppressive therapy for the past year, successfully treated by endoscopic full-thickness resection (EFTR). A 56-year-old woman presented with a 20-year history of postprandial left upper quadrant pain that had initially responded to proton pump inhibitors (PPIs) but became refractory over the past year. Repeated endoscopies revealed a gastric diverticulum with food impaction. She underwent EFTR for resection of the lesion. The postoperative course was uneventful, with no complications, and she recovered well. Postoperative histopathology confirmed a false gastric diverticulum lacking the muscular layer, with no evidence of neoplasia. At one month follow up, her abdominal pain had resolved, and endoscopy showed good healing of the resection site. This case suggests that EFTR is an effective treatment option for small, medication-refractory gastric diverticula. However, further studies are needed to define its optimal indications. Given the potential risk of coexisting neoplasia in false diverticula, more aggressive management and careful histopathological examination may be warranted.

## Introduction

Gastric diverticula are the rarest type of gastrointestinal diverticula, with a reported incidence of 0.013%–2.6% (1). No established management guidelines exist; most are incidental findings that require no treatment or only pharmacotherapy. Medication-refractory cases have conventionally been treated by laparoscopy, which is more invasive and has a longer recovery time than endoscopic resection. We report the case of a 56-year-old woman with a 20-year history of postprandial abdominal pain, which was initially well controlled with proton pump inhibitor (PPI) therapy but had worsened over the past year and became poorly responsive to PPIs. Physical examination was unremarkable, and routine laboratory tests showed no significant abnormalities. She underwent endoscopic full-thickness resection (EFTR) for the gastric diverticulum. The procedure was uneventful, without complications. To our knowledge, this is the first description of EFTR for a false gastric diverticulum. The case suggests that EFTR may be a feasible minimally invasive option for small, PPI-refractory gastric diverticula.

## Case presentation

**Figure 1:**
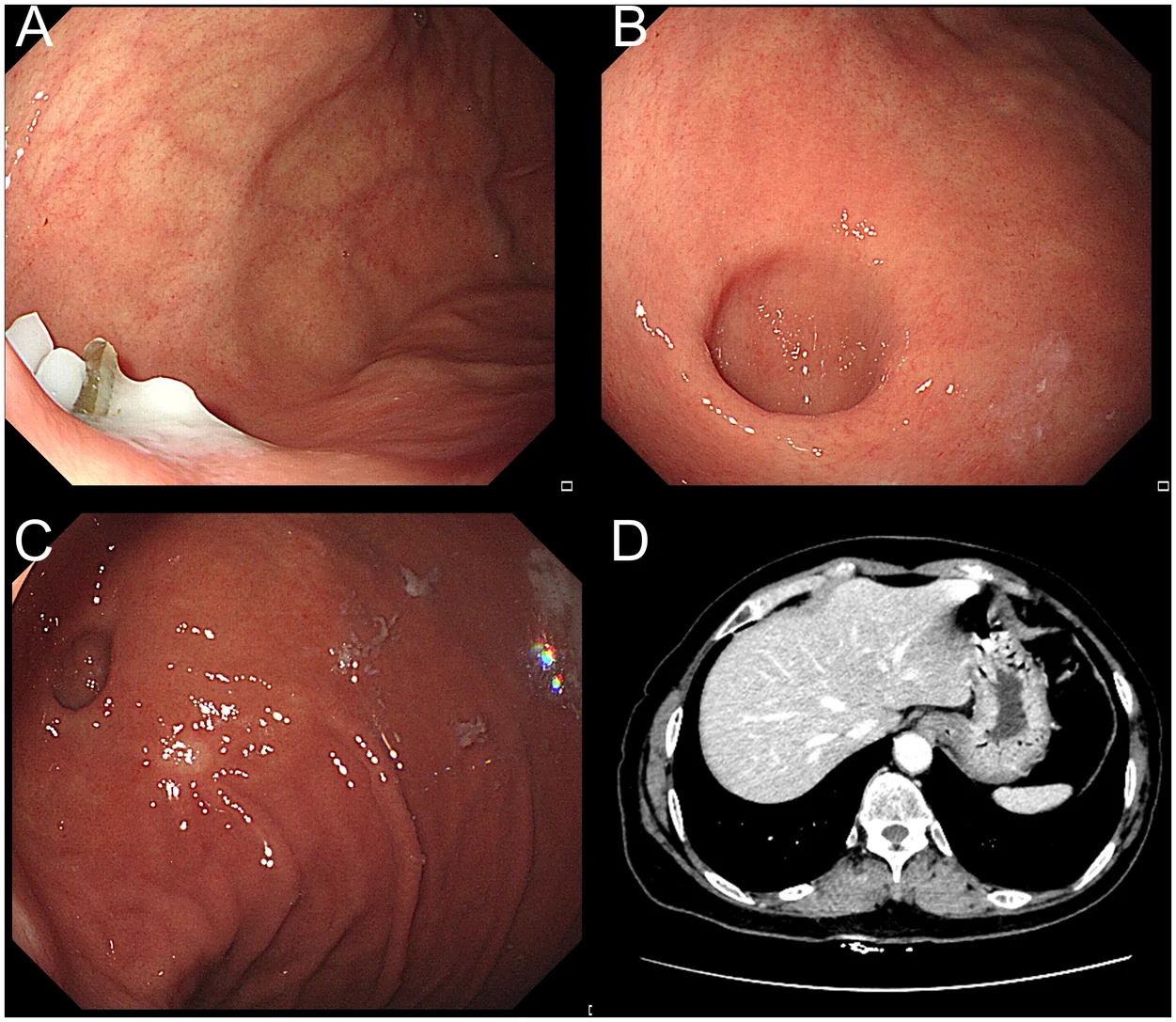
**(A)** a gastric diverticulum with food retention;**(B)** gastric fundus diverticulum. **(C)** A wider endoscopic view showing the diverticulum in the gastric fundus. **(D)** Contrast-enhanced abdominal CT image showing an exophytic nodule at the gastric fundus.

A 56-year-old woman was admitted with a 20-year history of recurrent left upper quadrant pain that had worsened over the preceding year. The pain was postprandial, radiated to the back, and partially responsive to empiric proton pump inhibitor (PPI) therapy, with prompt relapse upon discontinuation. The patient had previously responded well to PPIs, though prior esophagogastroduodenoscopy (EGD) had shown a gastric diverticulum with food retention that was managed expectantly. Over the past year, however, her symptoms had become refractory to escalating PPI therapy, accompanied by unintentional weight loss of 3 kg. Physical examination revealed no significant abnormalities. Her medical history included a prior cerebral infarction, for which she was not on any regular medication. Outpatient gastroscopy revealed a fundic diverticulum with retained food and chronic non-atrophic gastritis (Figure 1). The clinical diagnosis was a symptomatic gastric diverticulum.

Given the patient’s preference for a minimally invasive approach, she was admitted for endoscopic resection.

Routine preoperative evaluation revealed: total bile acids 13.8 µmol/L (reference range 0.0–10.0 µmol/L); stool analysis showed fungal spores and weakly positive occult blood; contrast-enhanced abdominal CT demonstrated an exophytic nodule at the gastric fundus, possibly a stromal tumor with necrosis; colonoscopy revealed no abnormalities in the visualized bowel segments aside from internal hemorrhoids. The remaining laboratory tests and examinations were unremarkable, and no contraindications to surgery were identified.

Differential diagnosis: The exophytic nodule at the gastric fundus demonstrated on preoperative contrast-enhanced CT(Figure 1D) required differentiation from submucosal tumors such as gastrointestinal stromal tumors (GISTs). GISTs are the most common gastric submucosal tumors, frequently located in the gastric fundus, and typically present as elevated lesions on endoscopy. However, in this patient, repeated preoperative endoscopies consistently showed a diverticulum with food impaction rather than a submucosal elevation. Moreover, a CT performed six months prior to admission had described a small saccular protrusion at the gastric fundus, suggestive of a diverticulum. Taken together, these findings suggest that the CT appearance of an “exophytic nodule” in the present study may have resulted from transient morphological changes caused by food impaction within the diverticulum, rather than representing a true neoplastic lesion. This interpretation was ultimately confirmed by postoperative histopathology, which revealed a false diverticulum with no neoplastic cells, thereby excluding GIST. Other differential considerations for fundic lesions—such as leiomyoma or extrinsic compression from adjacent organs (e.g., spleen or pancreatic tail)—were also considered but were essentially ruled out by the endoscopic findings and histopathological results.

**Figure 2:**
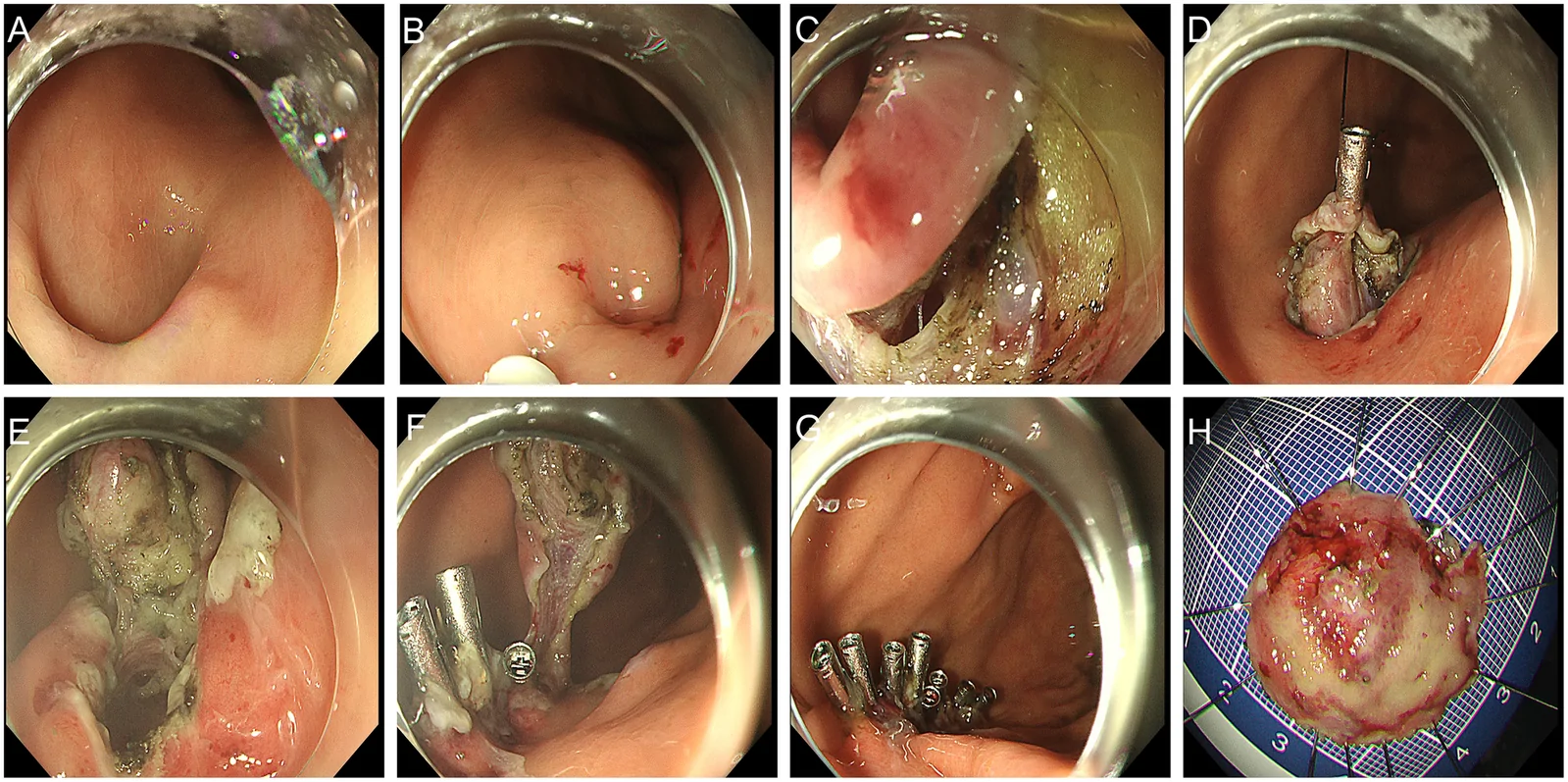
Intraoperative images of the procedure. **(A)** Gastric fundus diverticulum; **(B)** Mucosal incision with a Dual knife; **(C)** Full-thickness resection being performed;**(D)** Traction using dental floss and a hemoclip; **(E)** Muscularis propria perforation, approximately 2 cm in size; **(F)** Closure of the defect;**(G)** Completed closure of the defect; **(H)** Resected diverticulum specimen.

After a comprehensive preoperative assessment, the patient underwent EFTR. During the procedure (Figure 2), a 1.0-cm circular fundic diverticulum was identified. We injected a methylene blue-saline mixture around the lesion, then initiated full-thickness resection using a Dual knife with cap assistance, with traction provided by dental floss and a hemoclip. The resection was completed with an insulated-tip (IT) knife. Minimal bleeding occurred during the procedure, which was controlled with IT knife coagulation; no further active bleeding was observed. A 2.0-cm muscularis propria perforation was closed with eight type-C hemoclips and one type-F hemoclip. The specimen was retrieved and sent for histopathological examination. The patient had an uneventful recovery, with no evidence of subcutaneous emphysema.

Postoperative supportive care consisted of prophylactic cefuroxime, esomeprazole for acid suppression, temporary fasting, and intravenous fluids. Laboratory tests on postoperative day 1 showed a decreased eosinophil count of 0.01 × 10⁹/L (reference: 0.02–0.52 × 10⁹/L) and a percentage of 0.1% (reference: 0.4%–8.0%), with remaining complete blood count parameters unremarkable.

The patient tolerated a liquid diet on postoperative day 2 and was discharged in good condition on day 3.

Histopathology of the resected specimen demonstrated a 1.4 × 1.0 cm diverticulum with chronic inflammation, vascular ectasia, and submucosal fibrosis; no neoplasia was identified. The absence of muscularis propria confirmed the diagnosis of a false diverticulum.

**Figure 3:**
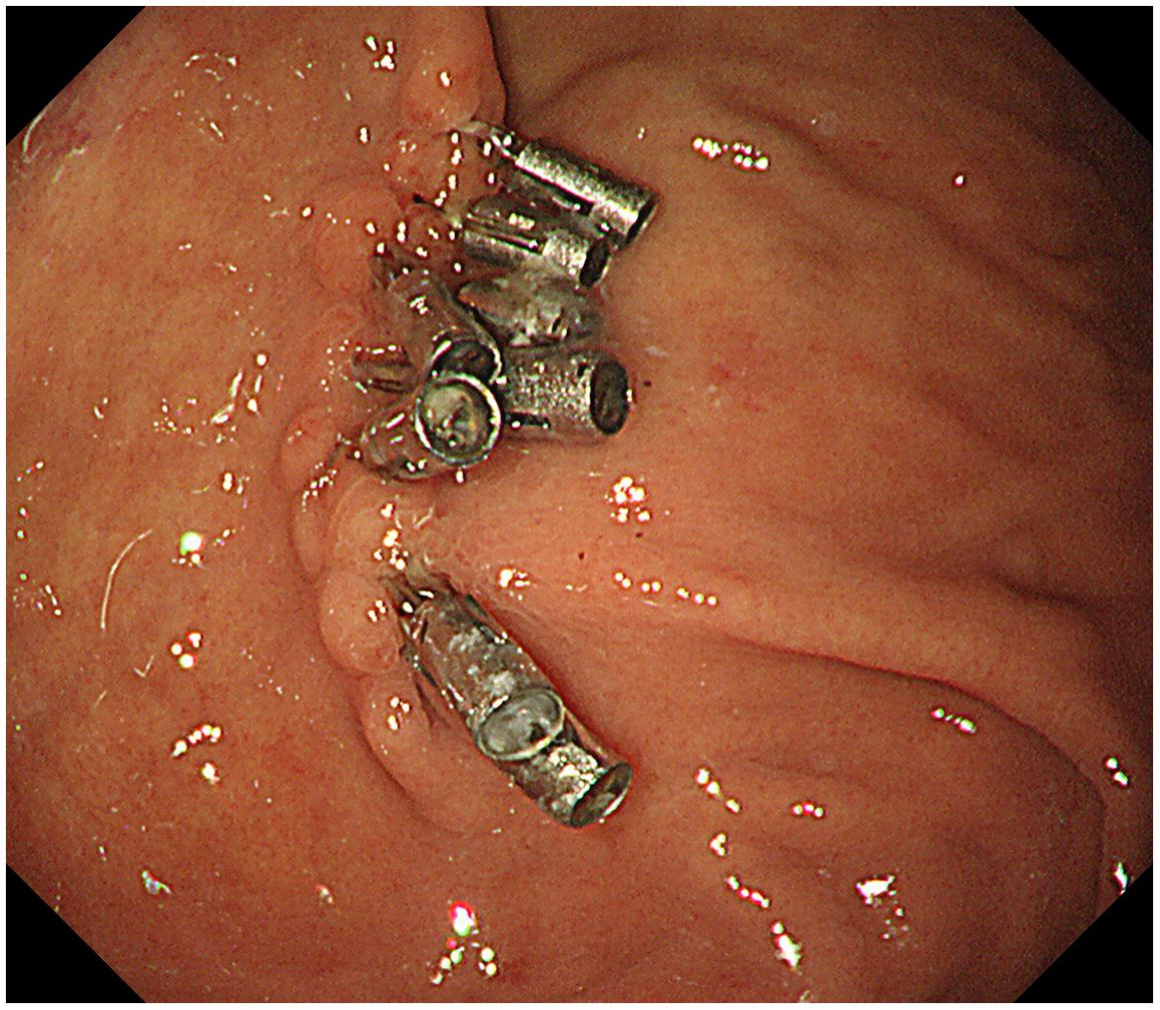
One-month follow-up endoscopy.

At the one-month follow-up, the patient reported substantial improvement in abdominal pain, and endoscopy showed good healing of the resection site (Figure 3). A telephone follow-up at 8 months post-procedure confirmed sustained symptom relief; she had discontinued PPIs one month after surgery, with no recurrence of symptoms. Long-term endoscopic follow-up images were not obtained, as the patient remained asymptomatic after surgery and routine repeat endoscopy was not performed. A timeline of the clinical course is provided in Table 1.

**Table 1:** Clinical timeline of symptoms, diagnosis, and treatment.

Dateline	Clinical timeline
2006–2024	The patient presented with abdominal pain, and gastroscopy revealed a gastric diverticulum. Her symptoms responded well to PPI therapy but recurred upon discontinuation.
2024–2025	The pain worsened, and endoscopy revealed a gastric diverticulum with food impaction. PPIs were administered but proved ineffective.
2025–12–20	The patient presented with abdominal pain and was admitted for elective endoscopic resection.
2025-12-22	The patient underwent EFTR.
2025-12-24	The patient started a liquid diet.
2025-12-25	The patient was discharged.
2026-1-29	At outpatient follow-up, endoscopy showed symptom improvement and satisfactory healing.

## Systematic review

This systematic review was conducted and reported in accordance with the Preferred Reporting Items for Systematic Reviews and Meta-Analyses (PRISMA) 2020 guidelines (2). A systematic literature search was performed in the PubMed database for studies published between January 1, 2000 and August 10, 2026. The search strategy combined Medical Subject Headings (MeSH) and free-text terms as follows: [“Diverticulum, Stomach”[Mesh] OR gastric diverticul*[Title/Abstract] OR stomach diverticul*[Title/Abstract]]. The search was restricted to English-language articles and human studies using PubMed filters. The time limit from 2000 to 2026 was set to capture contemporary clinical treatment practices following the development of minimally invasive techniques over the past two decades. No publication type restriction was applied in the initial search to avoid missing relevant case series that might not have been indexed as “case reports.” In addition, to compensate for potential omissions due to database indexing delays, we manually browsed relevant journal websites and performed a DOI search to supplement one recently published article not yet indexed in PubMed (Li et al., 2026). This article was classified under “Other sources” in the PRISMA 2020 flow diagram. The PRISMA flow diagram is presented in Supplementary Figure 1.

Studies were included if they met all of the following criteria: (1) definite diagnosis of gastric diverticulum by endoscopy, imaging, or surgery; (2) at least one specific therapeutic intervention; (3) reporting of treatment-related outcomes; and (4) case reports or case series. Studies were excluded if they had no specific treatment, if the diverticulum was not the primary lesion, if they were duplicate publications, if patient-level data could not be extracted, or if full text was unavailable.

The following information was extracted from each included study: first author, year of publication, patient age and sex, diverticulum type (true/false, as reported or classified by the authors), diverticulum location and size, presenting symptoms, treatment modality, clinical outcomes (e.g., symptom resolution, procedural success, complications), and duration of follow-up. For cases lacking histopathological confirmation of the type classification, if not reported in the original article, it was recorded as “not reported”; only when the reported location or etiology strongly supported a specific type was it recorded as “likely true” or “likely false.” All extracted data from the included studies are presented in tabular format (Supplementary Table 1) to facilitate comparison across cases.

Among the 36 included patients, surgical resection was the most frequently employed treatment modality, accounting for 28 cases (77.8%). Endoscopic intervention was performed in 2 cases (5.6%): one patient underwent endoscopic epinephrine injection combined with hemoclips for diverticular bleeding (thermal coagulation was avoided) (3) and the other underwent endoscopic diverticulectomy for a false gastric diverticulum (4). Conservative management with proton-pump inhibitors or dietary modification was used in 6 cases (16.7%). In summary, laparoscopic surgical resection remains the mainstay of treatment for symptomatic gastric diverticula, whereas endoscopic therapy has been reported only in sporadic cases, with EFTR being exceptionally rare in the current literature.

## Discussion

Gastric diverticula, defined as saccular outpouchings of the gastric wall, are the rarest type of gastrointestinal diverticula (1). They are divided into true and false types based on whether the wall contains all gastric layers. True diverticula are generally thought to be congenital, related to focal weakness or developmental defects of the musculature. False diverticula, by contrast, are mostly acquired. Their wall consists only of mucosa and submucosa, with no intact muscularis propria (1, 5).Their formation can be attributed to three main mechanisms: sustained intraluminal pressure (e.g., from gastric outlet obstruction), leading to herniation of mucosa and submucosa—the so-called pulsion diverticulum; structural wall damage (e.g., peptic ulcer, tumor infiltration, or postoperative weakness), creating a site vulnerable to herniation; and perigastric adhesions from chronic inflammation (e.g., pancreatitis or cholecystitis), which result in a traction diverticulum. No standardized management guidelines exist. Proton pump inhibitors are first-line for symptomatic patients, but those who are refractory may require surgical intervention. Earlier reviews recommended surgery for diverticula >4 cm (6), though this size-based threshold is not absolute: some 5-cm diverticula have been managed conservatively (7, 8), while smaller lesions have required surgery (9, 10).

Compared with the conventional laparoscopic management of medication refractory gastric diverticula, the present case demonstrates endoscopic resection as a new therapeutic option for small, PPI refractory gastric diverticula. EFTR is a deliberate perforation technique enabling full thickness excision. It is conventionally indicated for subepithelial tumors involving the muscularis propria or for epithelial neoplasia with significant submucosal fibrosis that cannot be safely removed by conventional endoscopic mucosal resection (EMR) or endoscopic submucosal dissection (ESD) (11).In our case, EFTR corrected the structural abnormality of a false gastric diverticulum, offering a less traumatic, faster recovering alternative to laparoscopic surgery via natural orifice.

Beyond technical feasibility, false diverticula carry specific risks. Although generally considered benign, they have been associated with malignancy (12–17). Oya et al. reported an adenocarcinoma arising within a gastric false diverticulum and suggested that persistent chronic inflammation (in the absence of *H. pylori*) may play a role in carcinogenesis (14). Bacha et al. likewise emphasized that false diverticula, particularly those in the prepyloric region, warrant adequate tissue sampling to exclude malignancy (5). In our patient, histopathology showed no neoplasia, but did reveal chronic inflammatory infiltration and submucosal fibrosis—changes that have been described in the setting of diverticular disease. Moreover, lacking the muscularis propria, false diverticula are structurally weaker than true diverticula, potentially predisposing them to complications such as hemorrhage or perforation (18). Thus, the potential for malignancy and the structural vulnerability of false diverticula warrant continued clinical awareness. The long-standing postprandial symptoms and repeated endoscopic evidence of food impaction in this case are consistent with a predominantly pulsion-related mechanism, although further investigation is needed to clarify the precise underlying mechanism. Whether false diverticula require more aggressive intervention than true diverticula remains to be determined.

The present case suggests that EFTR can be effective for small, symptomatic, PPI-refractory gastric diverticula, offering rapid recovery and a minimally invasive alternative to laparoscopic surgery. At the same time, the absence of a muscular layer in false diverticula warrants vigilance regarding potential complications and malignant transformation, and histopathological examination remains essential to exclude coexisting neoplasia. Larger, prospective studies are needed to refine patient selection and establish more definitive management protocols.

Recently, Li et al. reported endoscopic resection of a true gastric diverticulum (4). In contrast, our case presents the first application of endoscopic full-thickness resection (EFTR) for a medication-refractory false gastric diverticulum. The procedure was well tolerated; the patient resumed a diet on day 2, was discharged on day 3, and reported sustained symptom relief at eight months. These early results suggest that EFTR may serve as a minimally invasive alternative to laparoscopic surgery in selected cases, particularly for false diverticula with refractory symptoms. However, this is a single-center, single-case report without preoperative endoscopic ultrasound (EUS), which limits the generalizability of our findings. Larger studies are needed to better define patient selection and to standardize the use of EFTR in this setting.

The present case suggests that EFTR can be effective for small, symptomatic, PPI-refractory false gastric diverticula, offering rapid recovery and a minimally invasive alternative to laparoscopic surgery. At the same time, the absence of a muscular layer in false diverticula warrants vigilance regarding potential complications and malignant transformation, and histopathological examination remains essential to exclude coexisting neoplasia. Larger, prospective studies are needed to refine patient selection and establish more definitive management protocols, and to further compare EFTR with the recently reported endoscopic resection for true diverticula.

## Data Availability

The raw data supporting the conclusions of this article will be made available by the authors, without undue reservation.
