# Supplementary material for: Case Report: Endoscopic full-thickness resection using dual and IT knives for a false gastric diverticulum and systematic review
Source: Front Med (Lausanne). 2026 Sep 9;13:1938681. doi: 10.3389/fmed.2026.1938681 (PMC13597830; doi:10.3389/fmed.2026.1938681)
Supplement: Supplementary file 1 [file Image1.pdf]

Supplementary Material

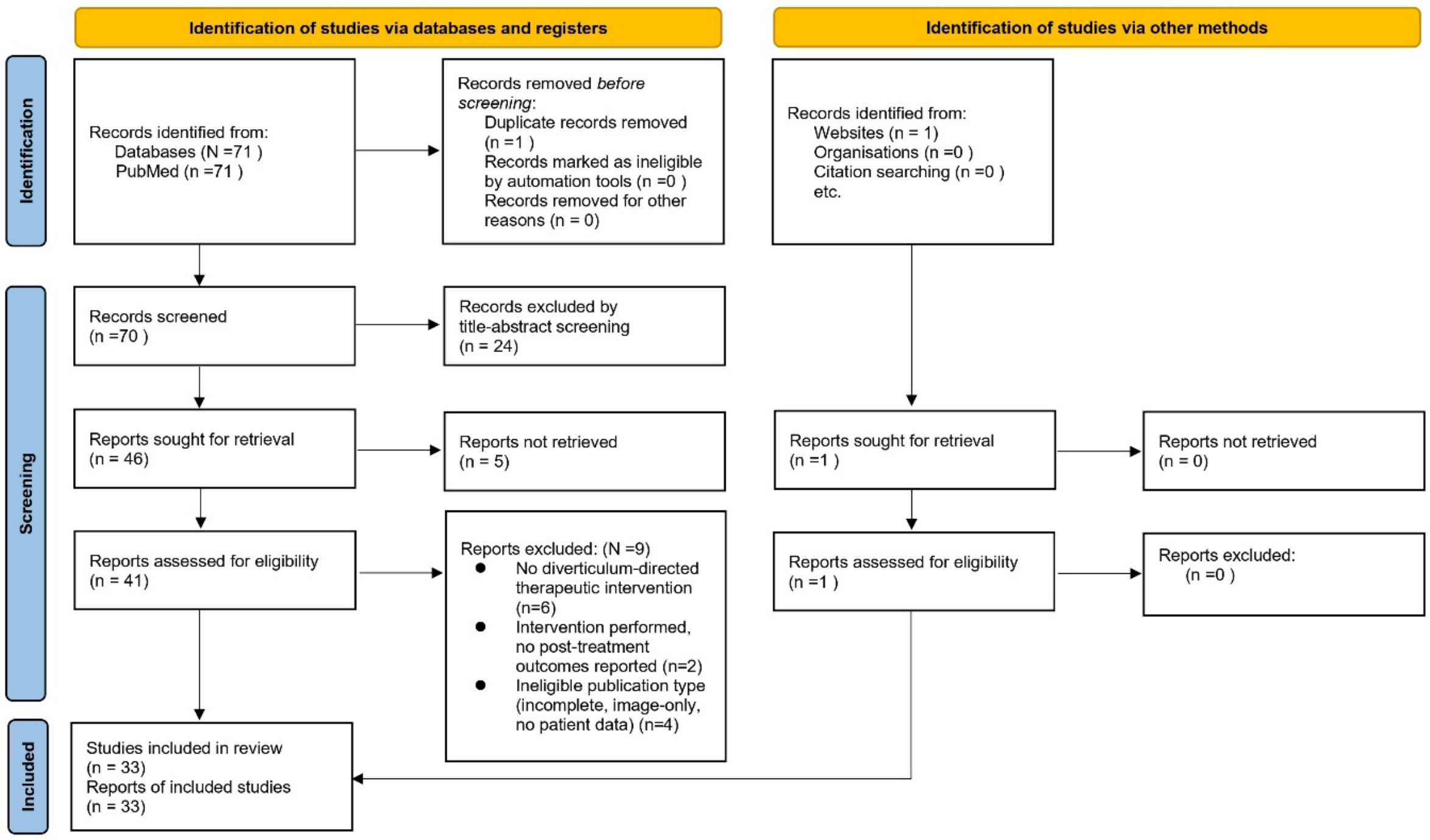

Supplementary Figure 1. PRISMA 2020 flow diagram of the study selection process.
