# Supplementary material for: Case Report: Endoscopic full-thickness resection using dual and IT knives for a false gastric diverticulum and systematic review
Source: Front Med (Lausanne). 2026 Sep 9;13:1938681. doi: 10.3389/fmed.2026.1938681 (PMC13597830; doi:10.3389/fmed.2026.1938681)
Supplement: Supplementary file 2 [file Table1.docx]

Supplementary Material

| No | First Author, Year | Age / Sex | Diverticulum location, size | Diverticulum type (histology / inference) | Main symptoms, complications | Therapeutic regimen | Post-operative / follow-up outcome | Follow-up duration |
| --- | --- | --- | --- | --- | --- | --- | --- | --- |
| 1 | Rodeberg, 2002 | 15 / F; 18 / M; 15 / F; 9 / M | P1: lesser-curvature gastric fundus, 5 cm distal to GEJ, posterior fundus. P2: prepyloric 8 mm diverticulum. P3: posterior fundus. P4: cardia | P1 true diverticulum with benign gastric mucosa, no heterotopic tissue. P2 true diverticulum with heterotopic pancreatic duct tissue. P3/P4 histology NR | Pediatric series: recurrent upper abdominal / left upper quadrant (LUQ )/ epigastric pain; nausea/vomiting or constipation in some; one with weight loss | P1 laparoscopic attempt converted to open diverticulectomy with GIA. P2 laparotomy excision plus pyloroplasty. P3 antacids/H2 blocker. P4 PPI | P1 pain-free; P2 symptom-free at last contact; P3 intermittent symptoms until unrelated death; P4 intermittent symptoms partially responsive to PPI | Long-term follow-up by chart/phone; P1 2 years; P2 last contact 1958; P3 until 1989; P4 recent phone interview |
| 2 | Gockel, 2004 | 47 / M | Large pouch in gastric fundus with hiatal hernia; size NR | Histology NR; true vs pseudodiverticulum not specified | Several years of reflux, heartburn, epigastric fullness and prominent burping; Barrett's esophagus | Argon plasma coagulation for Barrett's segment; PPI failure; laparoscopic Nissen fundoplication with simultaneous diverticulum excision using Endo-GIA | Symptoms resolved after surgery | NR |
| 3 | McKay, 2005 | 30 / F | Posterior cardia / fundus; 3.0 x 2.5 x 2.0 cm specimen | False / partial-wall: mucosa, muscularis mucosa, submucosa and serosa present, muscularis propria absent | Halitosis, sour taste, bloating, right-sided abdominal pain; food retention | Laparoscopic resection with Endo GIA stapler | Uneventful; no recurrent symptoms | 28 months |
| 4 | Kubiak, 2006 | 13 / F | Posterior fundus adjacent to left adrenal; 12 mm diameter; approx. 5 cm below cardia | Likely true by pediatric posterior fundal location; histology showed fundal gastric mucosa without inflammation or dysplasia | Two years of recurrent abdominal / left flank / epigastric pain with nausea and vomiting; symptoms no longer responded to medication | Laparoscopic resection at base using linear cutting stapler | Uneventful; discharged on full oral feeds Post‑Operative Day (POD)3; well and symptom-free | 12 months |
| 5 | Elliott, 2006 | 15 / M | Posterior fundus just below GEJ / left suprarenal region; 5 cm | Likely congenital / true by pediatric posterior fundal location; pathology showed gastric mucosa with patchy chronic inflammation, no heterotopic tissue | Left flank pressure / full feeling for 4 months, worse with eating and lying left; PPI failed; mimicked left suprarenal lesion | Open surgical excision through left subcostal incision; defect closed in two layers | Discharged POD4 tolerating regular diet; antireflux medication stopped; symptom-free | 1 year |
| 6 | Goitein, 2006 | 40 / F | Posterior proximal gastric pouch / cardia after Roux-en-Y gastric bypass; wide neck; approx. 1 x 3 cm, behind Roux limb | True diverticulum: all layers of gastric wall present; no staples on diverticulum surface, so not clearly created during pouch formation | Post-LRYGB nausea, dysphagia and intolerance to liquids; grade 1 esophagitis; no ulceration or bleeding | Laparoscopic resection assisted by intraoperative endoscopy; transected at neck with endoscopic linear stapler | Uneventful; discharged POD3 tolerating liquid diet; symptom-free | 9 months |
| 7 | Lopez, 2007 | 15 / F | Gastric fundus, inferior and posterior to GEJ; 2 cm | True / congenital: normal full-thickness gastric tissue | Five-year sharp epigastric pain worsened by food, with nausea/vomiting and prior rectal bleeding; PPI/H2 blocker/antacid/analgesic nonresponse | Laparoscopic five-port diverticulectomy using a linear cutting and stapling device | Uneventful; repeat EGD at 3 months showed scar; subsequently pain-free | 18 months |
| 8 | Donkervoort, 2006 | 45 / F | High posterior corpus-fundic region, 2 cm from GOJ; 5 cm endoscopic length, 5.5 x 4 x 2.5 cm specimen | Indeterminate: normal mucosa, thickened muscularis mucosae, thin and partly discontinuous muscularis propria | Severe foetor ex ore, nocturnal vomiting, food/contrast retention; PPI nonresponse | Laparoscopic EndoGIA resection | Uneventful recovery; foetor and nocturnal vomiting resolved | NR |
| 9 | Lajoie, 2008 | 51 / M | Posterior gastric wall 3 cm distal to GEJ; endoscopic bleeding diverticulum 1.5 cm; operative specimen approx. 2 x 1.3 x 1.4 cm high posterior cardia | Likely true by posterior cardia location; pathology benign fundic mucosa without ulceration or malignancy; layers NR | Fatigue and black stools for 3 days; anemia; acute hemorrhage from diverticulum | Endoscopic irrigation; epinephrine injection ineffective; India ink marking; surgery with laparotomy, anterior gastrotomy, gastroscopy, vessel ligation, amputation and oversewing | No further bleeding; discharged 5 days later | In-hospital follow-up; longer NR |
| 10 | Chen, 2008 | 67 / M | Posterolateral proximal stomach; 4 cm diverticulum on subsequent barium study | Histology NR; typical proximal gastric diverticulum | Melena for 4 days, Hb 85 g/L; exposed vessel in small ulcer on diverticular margin | Endoscopic epinephrine injection plus hemoclips; thermal coagulation avoided | Bleeding settled after endoscopic therapy | Subsequent barium study; longer NR |
| 11 | Mohan, 2010 | 58 / M | Fundus; size NR | NR | Upper abdominal pain and anorexia for 1 month; pain reproduced by probing/insufflation; no dysphagia, vomiting, hematemesis or melena | Proton-pump inhibitor therapy for 4 weeks | Symptoms improved after 4 weeks | 4 weeks |
| 12 | Tebala, 2010 | 46 / F | Posterior aspect of upper gastric fundus, about 4 cm from cardia; small pear-shaped pouch | True diverticulum: all layers normally represented | Epigastric pain and fullness; mimicked diaphragmatic / paraesophageal hernia | Laparoscopic 4-port resection with endoscopic stapler; stump anchored to left crus | Uneventful; POD3 swallow showed no leak; discharged POD4; well and symptom-free | >2 months |
| 13 | Gurvits, 2010 | 33 / F | Posterior gastric fundus / juxtacardiac diverticulum; 5 cm with food debris | Likely congenital / true by juxtacardiac posterior fundus location; histology NR | Hematemesis for 2 days and postprandial upper abdominal discomfort; recent diverticular bleeding; no active vessel or mass on OGD | Discharged on proton-pump inhibitor | Did well on follow-up | NR |
| 14 | Lhewa, 2011 | 14 / F | Prepyloric / gastric antrum; 2 cm deep pouch with approx. 8 mm orifice | Likely acquired / traction-type intramural diverticulum with heterotopic pancreatic parenchyma; antral location supports acquired etiology | Persistent epigastric pain for 2 months; eosinophilic gastritis; symptoms persisted on omeprazole | Surgery after omeprazole failure | Pain initially decreased after surgery but returned, possibly related to eosinophilic gastritis | NR |
| 15 | Oya, 2012 | 63 / F | Upper gastric body, greater curvature; lesion about 10 mm initially, enlarged to 25 mm | Intramural pseudo-/partial diverticulum with non-neoplastic mucosa and intramucosal adenocarcinoma with minimal invasion | Long-standing submucosal tumor followed for 14 years; papillary lesions and atypical epithelium suggesting adenocarcinoma | Laparoscopic wedge resection | Postoperative outcome NR | Preoperative lesion follow-up about 14 years; postoperative NR |
| 16 | Eitzen, 2012 | 44 / F | Fundus; single large diverticulum, 5 cm | Likely congenital / true by fundal location; histology NR | Recurrent abdominal pain and bloating, altered bowel habits, rectal bleeding episodes, nocturnal reflux-like chest pain / dyspepsia | Non-surgical management with proton-pump inhibitor | Currently well with symptoms managed by PPI | NR |
| 17 | DuBois, 2012 | 45 / M | Fundus / posterior fundus; 3 cm | Likely true by fundal/posterior location; pathology NR | Four months of dysphagia, regurgitation and abdominal pain | Laparoscopic gastric diverticulectomy using stapler with staple-line reinforcement and leak test | Uneventful; regular diet POD2, discharged POD3; no symptoms at first follow-up | First follow-up, duration NR |
| 18 | Carbonero-Celis, 2013 | 22 months / NR | Antrum near pylorus; 1 cm | Likely acquired / pseudodiverticulum associated with heterotopic pancreas; mature pancreatic tissue in submucosa | Hematemesis (coffee-ground), melena and fever; heterotopic pancreas | Omeprazole followed by laparoscopic resection | Favorable evolution; asymptomatic | 1 year |
| 19 | Globke, 2013 | 46 / F | Subcardial / dorsal gastric wall; 35 mm aperture | True / congenital: full-thickness stomach specimen, slight chronic inflammation, otherwise normal mucosa | Acute exacerbation of abdominal pain, recurrent nausea/vomiting, reflux symptoms, fetor ex ore, rapid 16 kg weight loss; mimicked left adrenal mass | Endoscopic-laparoscopic rendezvous resection with EchelonFlex60 Endopath stapler | Fetor and reflux ceased immediately; contrast swallow showed patent suture; discharged after POD5 study | POD5 imaging; longer NR |
| 20 | Marano, 2013 | 51 / F; 49 / F | Posterior fundus; 5 cm and 8 cm | Likely true by posterior fundal location; histology confirmed normal mucosa, layers NR | Epigastralgia and upper abdominal tenderness; no weight loss, hematemesis, melena, vomiting or bowel dysfunction | Laparoscopic resection under intraoperative endoscopic control with EndoGIA | Uneventful; no complications; asymptomatic with normal radiographic/endoscopic and functional exams | 6 months |
| 21 | Pietrzak, 2013 | 18 / F | Antrum; abscess immediately caudal/near pylorus; size NR | Likely acquired; pathology showed diverticulum with diverticulitis and heterotopic pancreas | Recurrent abdominal pain; recurrent intra-abdominal abscess formation | Percutaneous drainage and antibiotics initially, then laparoscopic partial gastrectomy | No postoperative complications | NR |
| 22 | Ho, 2013 | 42 / M | Greater curvature; 4 cm outpouching with coffee-ground material / food residue | False diverticulum: gastric mucosal herniation without muscular coating | Epigastric pain and tarry stools for 3 days, anemia, free gas; prior bleeding and perforation | Initially conservative management, then laparoscopic stapled diverticulectomy | Postoperative outcome NR | 2-week diagnostic EGD before surgery; postoperative follow-up NR |
| 23 | Zelisko, 2014 | 54 / M; 58 / M | Posterior fundus; case 1 wide-mouth 4 cm diverticulum 2 cm from EGJ; case 2 3.7 cm on CT / 5 cm intraop, 3 cm from GEJ | Likely true by posterior fundal location; histology NR | Case 1: recurrent nausea/vomiting, bowel change, periumbilical pain, postprandial fullness, distention, foul eructation. Case 2: epigastric/LUQ pain | Laparoscopic partial gastrectomy/diverticulectomy with stapled base oversewn; case 2 also hiatal hernia repair and adrenalectomy work-up | Case 1 complete symptom resolution. Case 2 discharged POD3, then port-site closure-related SBO treated surgically; later no pain and regular diet | 1 month; 3 months |
| 24 | Surve, 2016 | Middle-aged / F | Fundus after gastric imbrication; large diverticulum with 3 cm neck; 1 cm from angle of His | Acquired / false inferred from prior gastric imbrication; histology NR | Chronic nausea, vomiting and LUQ abdominal pain 5 years after laparoscopic gastric imbrication; weight regain | Conversion to laparoscopic sleeve gastrectomy with diverticulum resection | Discharged POD3 without nausea/vomiting; symptom resolution reported | POD3 discharge; longer follow-up NR |
| 25 | Gravito-Soares, 2018 | 54 / M | Gastric fundus; 36 x 20 mm on endoscopy; MRI mass behind stomach 37 x 18 mm with air-fluid level | Likely congenital / true by solitary fundal location; histology NR | Nonspecific upper abdominal pain; discomfort reproduced by insufflating diverticulum | One month of PPI plus irritant-free diet low in acidic, spicy, highly salted and high-fat foods | Became asymptomatic after 1 month; no symptomatic relapse | 2 years |
| 26 | Bacha, 2019 | 69 / M | Pre-pyloric / antral region; 1 cm diverticulum with adjacent 70 x 50 mm wall thickening (71 x 35 mm on CT) | False pre-pyloric diverticulum: mucosal outpouching extending into muscularis, marked inflammation/fibrosis, adjacent vascular hamartoma; no malignancy | Diffuse abdominal pain, postprandial vomiting, severe gastric obstruction, marked weight loss, suspected malignancy | Exploratory laparotomy and antrectomy | Uneventful; discharged day 7; general condition improved with 5 kg weight gain | 6 months |
| 27 | Brucchi, 2023 | 69 / F | Gastric fundus / posterior wall; 28 x 38 mm on CT, 5 x 3 cm pathology specimen | True / congenital: pathology showed diverticulum composed of all layers of gastric wall | Persistent epigastric pain, retrosternal pain radiating posteriorly, nausea, bloating, weight loss from 78 to 70 kg; PPI failure | Laparoscopic complete isolation and stapled diverticulum excision with Signia stapler (60 mm plus 40 mm reloads); POD3 Upper Gastrointestinal (UGI) swallow | POD3 UGI showed no leak, preserved peristalsis and normal duodenal transit; discharged POD4; no GI symptoms or wound infection | 30 days |
| 28 | Sleiman, 2025 | 28 / F | Posterior fundus; 1.5 cm | Likely true / congenital by posterior fundal location; pathology confirmed fundal gastric diverticulum, layers NR | Morning emesis; PPI-refractory dyspepsia and epigastric pain; mild GERD; hepatic adenoma incidental | Laparoscopic proximal partial gastrectomy/diverticulectomy with endoscopic linear stapler | No complications; discharged POD2; asymptomatic; no residual/recurrent diverticulum on follow-up imaging/endoscopy | 1 month, 3 months and 1 year |
| 29 | Pazin, 2025 | 64 / F | Fundus / posterior stomach wall in close contact with spleen and left adrenal gland; 40 x 30 x 47 mm on CT | Congenital / true stomach diverticulum by report; resected wall was gastric wall, mucosa without metaplasia, dysplasia or inflammation | Two years of epigastric / left subcostal pain radiating to back, nocturnal reflux attacks and cough; partial PPI response then recurrence; mimicked hiatal hernia | Robotic diverticulum resection with 60 mm robotic linear endostapler, oversewn resection line; anterior hiatoplasty also performed | Uncomplicated; discharged POD4; wounds healed; off PPI by first check; preoperative symptoms completely disappeared | POD12 visit and 2-month telephone follow-up |
| 30 | Li J, 2026 | 50 / M | Posterior gastric wall, close/adherent to left adrenal region; 3 x 2 cm on MRI, approx. 2.5 cm intraoperatively | Hemorrhagic gastric diverticulum; resected specimen contained clotted blood; histopathology consistent with gastric diverticulum; true/false not specified | Upper abdominal discomfort, gastric stasis, occasional black stools; mimicked left adrenal tumor / pheochromocytoma | Planned posterior laparoscopic adrenal exploration; intraoperative identification of GD followed by partial gastrectomy at diverticular base with laparoscopic stapler; fasting and parenteral nutrition postoperatively | Discharged 1 week postoperatively; 1-month CT showed resolution of suspected adrenal mass; bloating and pain resolved | 1 month |
| 31 | Li M, 2026 | 60 / M | Gastric fundus; 20 mm orifice | Congenital / true supported by full-thickness gastric tissue with mild chronic inflammation and erosive change | Recurrent acid reflux, belching and chronic abdominal pain | Endoscopic diverticulectomy / full-thickness circumferential resection using Dual Knife/IT knife, traction, coagulation and clip closure | Symptoms resolved promptly; POD7 barium study showed well-healed resection margin; discharged without adverse events | POD7 barium study; longer follow-up NR |

Supplementary Table 1. Summary of clinical characteristics, treatment modalities, and outcomes of 36 patients with gastric diverticulum from 33 included case reports and case series.
